# Real-world clinical course of HTLV-1-associated myelopathy/tropical spastic paraparesis (HAM/TSP) in Japan

**DOI:** 10.1186/s13023-019-1212-4

**Published:** 2019-10-21

**Authors:** Shuntaro Tsutsumi, Tomoo Sato, Naoko Yagishita, Junji Yamauchi, Natsumi Araya, Daisuke Hasegawa, Misako Nagasaka, Ariella L. G. Coler-Reilly, Eisuke Inoue, Ayako Takata, Yoshihisa Yamano

**Affiliations:** 1 Department of Advanced Medical Innovation, St. Marianna University Graduate School of Medicine, 2-16-1 Sugao, Miyamae-ku, Kawasaki, Kanagawa 2168512 Japan; 2 Department of Rare Diseases Research, Institute of Medical Science, St. Marianna University School of Medicine, 2-16-1 Sugao, Miyamae-ku, Kawasaki, Kanagawa 2168512 Japan; 3 Department of Oncology, Karmanos Cancer Institute, Wayne State University, Detroit, MI USA; 4 Medical Informatics, St. Marianna University School of Medicine, Kawasaki, Japan; 5 Department of Preventive Medicine, St. Marianna University School of Medicine, Kawasaki, Japan

**Keywords:** HTLV-1, HAM/TSP, Historical control, Real-world data

## Abstract

**Background:**

As human T-cell leukemia virus type 1 (HTLV-1)-associated myelopathy/tropical spastic paraparesis (HAM/TSP) is a rare chronic neurological disease, large scale studies to collect continuous clinical data have been difficult to conduct. Therefore, the incidence of comorbidities and drug utilization data remain unknown. When conducting trials to develop new drugs in rare disease such as HAM/TSP, historical control data obtained from registry studies would be useful, as cohorts in rare disease tend to be small. Long-term follow-up of patients with a chronic disease can also be challenging. In this study, we addressed the following two goals using registry data on patients (*n* = 486) enrolled in the Japanese HAM/TSP patient registry “HAM-net” from 2012 to 2016: 1) to clarify the epidemiological information of HAM/TSP such as the incidence of comorbidities and drug utilization and 2) to provide the real-world data on changes in lower limb motor dysfunction.

**Results:**

In HAM-net-registered patients, common comorbidities were fractures, herpes zoster, and uveitis, with incidences of 55.5, 10.4, and 6.5, respectively, per 1000 person-years. Every year, oral steroid treatment was administered in 48.2–50.7% of the HAM-net-registered patients and interferon-α treatment was used in 2.6–3.5% of patients. The median dose of oral prednisolone was low at 5.0 mg/day. The incidence of fractures and herpes zoster tended to be higher in the steroid-treated group than in the untreated group (fractures: 61.0 vs. 48.3, herpes zoster: 12.7 vs. 8.8, per 1000 person-years). The analysis of chronological change in Osame motor disability score (OMDS) indicated that the mean change in OMDS was + 0.20 [95% confidence intervals (CI): 0.14–0.25] per year in the one-year observation group (*n* = 346) and + 0.57 (95% CI: 0.42–0.73) over four years in the four-year observation group (*n* = 148). Significant deterioration of OMDS was noted in all subgroups with varying steroid use status.

**Conclusions:**

This study revealed the incidence of comorbidities and drug utilization data in patients with HAM/TSP using registry data. Furthermore, this study provided real-world data on chronological changes in lower limb motor dysfunction in patients with HAM/TSP, indicating the utility of these data as historical controls.

## Background

Human T-cell leukemia virus type 1 (HTLV-1)-associated myelopathy/tropical spastic paraparesis (HAM/TSP) is a neuroinflammatory disease that develops in a small percentage (0.25–3.8%) of those infected with HTLV-1 [1, 2]. These patients usually experience a serious QOL decline due to gait disturbance, urinary dysfunction, as well as numbness and pain in their lower limbs. Generally, in Japan, HAM/TSP is treated with corticosteroids and interferon-α [2]. However, owing to insufficient efficacies and side effects, there is an urgent need to develop new treatments.

Nevertheless, it may be difficult to conduct controlled clinical trials because of the limited number of patients. In addition, even if the number of participants were to be sufficient, in a chronic disease such as HAM/TSP that is known to progress slowly over several years, there would be a serious ethical concern in setting up a control group that would be followed up on placebo alone for a prolonged period of time. In order to address these issues, the use of historical controls employing existing research data, such as from registry studies, can be constructive [3, 4]. Detailed and accurate data could enhance the feasibility of clinical trials and enable long-term evaluation of drug efficacy leading to more efficient development of treatment strategies.

The Osame motor disability score (OMDS, assessed on a scale from 0 to 13, Table 1) has been used frequently as the primary endpoint of HAM/TSP clinical trials [5, 6], and there have been several reports on the clinical course of HAM/TSP motor disability [7–9]. One example is a UK study reporting that the median times from disease onset to dependency on a unilateral walking aid and subsequently, a wheelchair, were 11 and 18 years, respectively [7]. A study based in Martinique reported a median duration of 6 years from onset to use of a unilateral walking aid, 13 years to a bilateral walking aid, and 21 years to wheelchair-dependency [8]. We found a median of 8 years from the onset of motor symptoms to unilateral support, 12.5 years to bilateral support, and 18 years to gait inability [9]. However, these observations were all based on retrospective studies, and the influences of treatment are unclear. Therefore, it is necessary to investigate the clinical course of HAM/TSP prospectively and to consider the treatments utilized.

**Table 1:** Osame motor disability score

Grade	Motor disability
0	No walking or running abnormalities
1	Normal gait but runs slowly
2	Abnormal gait (stumbling, stiffness)
3	Unable to run
4	Needs handrail to climb stairs
5	Needs a cane (unilateral support) to walk
6	Needs bilateral support to walk
7	Can walk 5–10 m with bilateral support
8	Can walk 1–5 m with bilateral support
9	Cannot walk, but able to crawl
10	Cannot crawl, but able to move using arms
11	Cannot move around, but able to turn over in bed
12	Cannot turn over in bed
13	Cannot even move toes

In rare diseases such as HAM/TSP, a patient registry system could be useful to prospectively collect information from as many patients as possible. We started the operation of the nationwide HAM/TSP patient registry “HAM-net” in Japan in 2012, and have already been able to report demographic and clinical features of patients with HAM/TSP [9, 10]. In Martinique, a patient registry was used to determine the incidence of HAM/TSP [11]. However, to our knowledge, no other HAM/TSP studies have employed such a patient registry approach. Indeed, many of the HAM/TSP reports that collected patient information were cross-sectional or retrospective studies. HAM/TSP patient data on the incidence rates of comorbidities and/or steroid-related complications, types of treatment and their continuation rates require prospective and continuous information collection.

The primary goal of this study was to identify important epidemiological information regarding HAM/TSP. This study focused on the incidence of comorbidities/steroid-related complications, and treatment continuation rates using HAM-net. The secondary purpose of this study was to provide data that could be used as historical controls by prospectively following the chronological changes in lower limb motor dysfunction in patients with HAM/TSP.

Using information obtained from a set of 486 HAM/TSP patients registered in “HAM-net” from 2012 to 2016, we examined the relevant epidemiological information, including patient characteristics, prevalence/incidence of comorbidities/steroid-related complications, and drug utilization. Next, we set up a new analysis set excluding patients with factors that may affect lower limb motor function and investigated patient characteristics and the time course of the OMDS in the new analysis set and four subgroups classified by their treatment condition.

## Methods

### Study design and patient registry system

This study was based on information from a Japanese HAM/TSP patient registry called “HAM-net.” This database was established to collect not only retrospective information such as medical and treatment histories, but also cross-sectional/prospective data such as clinical course and treatment status (UMIN trial number: UMIN000028400) [9]. The registered patients were those who met the two criteria of having a confirmed HAM/TSP diagnosis and having provided informed consent. Each subject was interviewed annually via telephone by a nurse or clinical research coordinator from the HAM-net study office. The interview phone script included patient characteristics, family and medical history, comorbidities, living backgrounds, living conditions, HAM/TSP symptoms, treatment history, and treatment status.

### Analysis sets to obtain epidemiological information

**Fig. 1:**
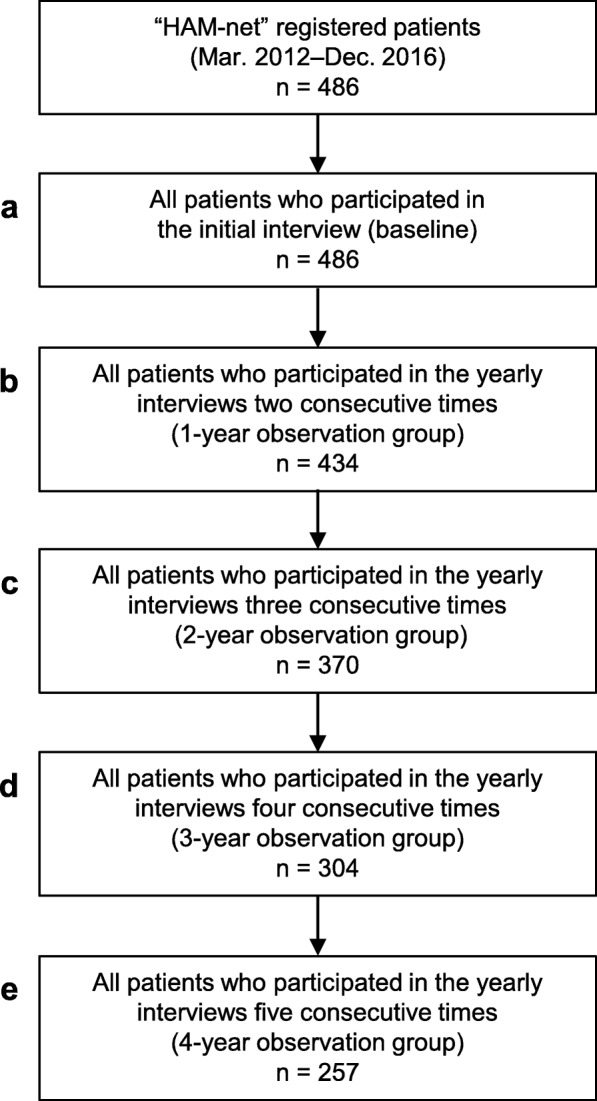
Flowchart showing analysis sets to conduct a fact-finding survey for HAM/TSP. The entire analysis set in this study consisted of 486 patients enrolled sequentially in the HAM/TSP patient registry “HAM-net” from March 2012 to December 2016. All 486 patients received an initial telephone interview just after registration (**a**). Information obtained from this interview was considered as baseline. Subsequently, we conducted annual interviews. We regarded the 434 patients who received an annual interview for two consecutive years as a one-year observation group (**b**). Likewise, we considered the 370 patients interviewed annually for three consecutive years as a two-year observation group (**c**). The three-year observation and the four-year observation groups consisted of 304 (**d**) and 257 patients (**e**), respectively. See Methods for details

Our entire analysis set consisted of 486 patients enrolled sequentially in HAM-net from March 2012 to December 2016 (Fig. 1). All subjects received the initial telephone interview just after registration (Fig. 1a). The HAM-net study office confirmed that all patients were at least 20 years old, diagnosed with HAM/TSP by their physician, and more than a year had passed since onset. The 486 patients who received the initial telephone interview received up to five annual telephone interviews over an observation period of up to four years. The data cut off for this study was December 2016. Therefore, patients who were enrolled in 2016 received only the initial interview, and although they are included in the 486-patient group (Fig. 1a), they were excluded from subsequent patient cohorts which required follow-up time (Fig. 1b–e). All 257 patients in the 4-year observation group (Fig. 1e) who received five consecutive interviews were enrolled in 2012 and were included in all groups shown in Fig. 1a–e. We included a total of 486 patients (Fig. 1a) in the analyses of patient characteristics, treatment status at the time of the initial interview, and the prevalence of comorbidities.

The 434 patients (Fig. 1b) who were observed for at least one year were included in the analyses of incidence rates of comorbidities and steroid-related complications. For the analysis of incidence of steroid-related complications by steroid treatment status, the patients were divided into three patient subgroups: 185 patients who had never received steroid therapy during the observation period (untreated group), 225 patients who received steroid treatment at least one time during the observation period (steroid group), and 181 patients who received steroid treatment continuously from among the above 225 patients (continued steroid group).

### Analysis sets for evaluating lower limb motor function

**Fig. 2:**
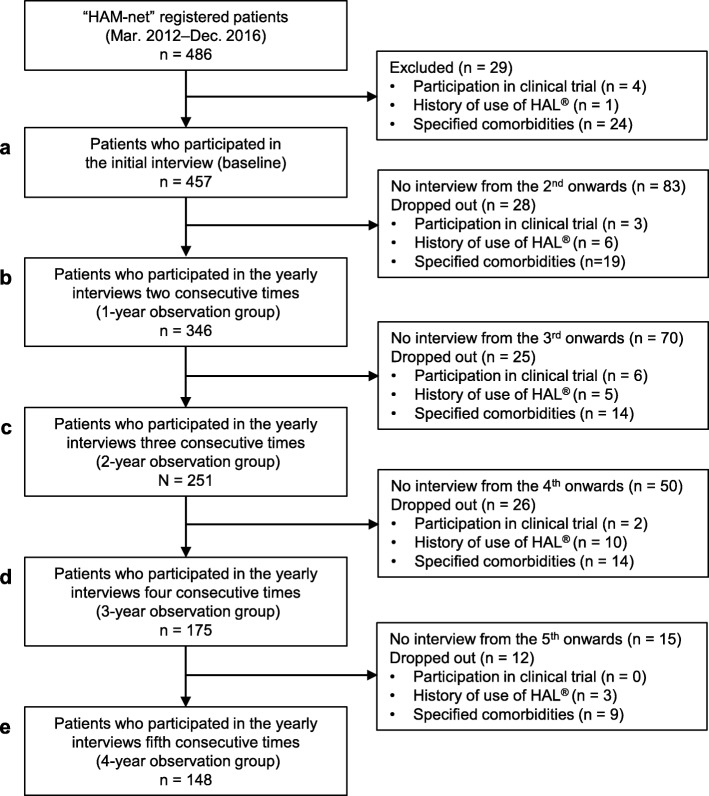
Flowchart for showing analysis sets to assess motor function in HAM/TSP (analysis set 2). To examine the chronological change of OMDS in patients with HAM/TSP, we excluded those who had factors that could affect the evaluation of lower limb motor function from 486 patients using specific exclusion/dropout criteria (see Methods for details). This cohort is referred to as “analysis set 2” in this paper. Furthermore, to accurately evaluate yearly changes in OMDS, we limited “analysis set 2” to patients whose interval between each survey date fell within 275–455 days (365 ± 90 days). The right half of this chart shows the number of patients who were excluded at each step and the reasons why. After being excluded by the criteria, there were 457 patients at the time of the initial interview (**a**). We used both the one-year observation group (*n* = 346, **b**) andthe four-year observation group (*n* = 148, **e**) for analysis. The two-year observation and the three-year observation groups consisted of 251 (**c**) and 175 patients (**d**), respectively

As shown in Fig. 2, the analysis sets for evaluating the chronological change of motor dysfunction in the lower limbs of patients with HAM/TSP (“analysis set 2”) consisted of patients who remained after excluding those who met the exclusion/dropout criteria:

A. Participants in the following clinical trials:

Phase I & IIa trials on anti-CCR4 antibody KW-0761 (UMIN trial number: UMIN000012655), Phase III trial on anti-CCR4 antibody KW-0761 (ClinicalTrials.gov Identifier: NCT03191526), Phase IIb trial on steroids (UMIN000023798, UMIN000024085, and UMIN000024086), and NCY-2001 clinical trial on Robot Suit HAL® (Hybrid Assistive Limb) (JMACCT ID: JMA-IIA00204 and JMA-IIA00257)

B. Patients who have undergone treatment with Robot Suit HAL®
C. Patients with any of the following serious comorbidities:

adult T-cell leukemia-lymphoma, active cancer, active tuberculosis, paralysis after stroke, Parkinsonian syndrome, rheumatoid arthritis, dementia, psychiatric disorder, and bone fracture that affects the ability to walk.

To accurately evaluate yearly changes in OMDS, the “analysis set 2” only included patients whose interval between each survey date fell within 365 ± 90 days. To clarify the influence of steroids on changes in OMDS, the analysis sets were classified into four subgroups: current steroid use, steroid-history, untreated, and miscellaneous (Table 2). Allocations into these subgroups were based on information regarding the following three parameters: history of steroid use, steroid use at the time of the initial interview, and steroid use between the initial interview and the final interview. The sub-analysis focused on patients having OMDS between 3 and 6. These are individuals who at the time of the initial interview could walk for ≥10 m with or without walking support and also have room for improvement in motor function in the lower limbs (Additional file 1: Figure S1).

**Table 2:** Four subgroups classified by treatment conditions

	History of steroid use	Steroid use at the time of the initial interview	Steroid use from the initial interview onwards
Steroid group	Yes	Yes	Yes
Steroid-history group	Yes	No	No
Untreated group	No	No	No
Miscellaneous group	All combinations other than the above		

### Specific items from the “HAM-net” registry

The data from HAM-net used in this study consisted of information obtained from three different periods:

A. *Retrospective data collected at the time of the initial interview*

Age at onset, medical history, treatment history (oral steroid therapy, steroid pulse therapy, interferon-α treatment), age at which each OMDS was reached, rapid progressor status. Rapid progressor was defined as patients with progression to OMDS grade ≥ 5 within 2 years after the onset of motor symptoms as described previously [10].

B. *Cross-sectional data at the time of the initial interview*

Age at baseline, gender, baseline OMDS, treatment status (oral steroid therapy, steroid pulse therapy, interferon-α treatment), dose of steroids, and comorbidities, as listed following [C].

C. *Prospective data surveyed annually after the initial interview*

OMDS, treatment history for one year from the time of last interview (oral steroid therapy, steroid pulse therapy, interferon-α treatment), and new-onset comorbidities listed as follows: for [B] and [C], the comorbitities were uveitis, Sjogren’s syndrome, rheumatoid arthritis, all bone fractures, herpes zoster, interstitial pneumonia, tuberculosis, diabetes mellitus, cataracts, and glaucoma. All bone fractures, herpes zoster, diabetes mellitus, cataracts, and glaucoma were classified as steroid-related complications.

### Calculations and statistical analysis

Each incidence rate of comorbidities and steroid-related complications per 1000 person-years was calculated using the number of new-onset patients between 2012 and 2016 as the numerator and the total years of follow-up between 2012 and 2016 as the denominator. The corresponding 95% confidence intervals (CI) were calculated using the Poisson distribution. We used the chi-square test to determine the independence of the nominal scale. One-way analysis of variance was used to compare the mean values of ≥3 groups. The Tukey post hoc test was used for multiple comparisons. Paired t-tests were used to analyze chronological changes in OMDS. Statistical analyses were performed using IBM SPSS Statistics Version 22 (IBM Corp. Armonk, NY, United States) or R version 3.4.2 (R Foundation for Statistical Computing, Vienna, Austria). All *p*-values were two-tailed, and the threshold of significance was set at 0.05.

## Results

### Patient characteristics, prevalence and incidence of comorbidities

We first investigated the characteristics and the prevalence of comorbidities at the time of the initial interview for all 486 patients enrolled in the HAM-net (Table 3). The age at the time of the initial interview was 62.0 ± 10.7 years, the age at onset was 44.8 ± 14.9 years, the disease duration was 16.1 ± 11.3 years, and the baseline OMDS was 5.7 ± 2.3 (all shown as mean ± standard deviation). The proportion of women and rapid progressors were 74.7 and 19.8%, respectively. At the time of the initial interview, the comorbidities with high prevalence were uveitis (7.6%), Sjogren’s syndrome (3.7%), and rheumatoid arthritis (2.7%).

**Table 3:** Patient characteristics and prevalence of comorbidities in HAM-net-registered patients

	All the HAM-net-registered patients (*n* = 486)
Sex: Female	363 (74.7%)
Age at baseline (year)^a^	62.0 ± 10.7
Age at onset (year)^a^	44.8 ± 14.9
Disease duration^a^ (time from onset to the baseline)	16.1 ± 11.3
Baseline OMDS^a^	5.7 ± 2.3
Rapid progressors^b^	96 (19.8%)
Comorbidities	Prevalence
Uveitis	37 (7.6%)
Sjogren’s syndrome	18 (3.7%)
Rheumatoid arthritis	13 (2.7%)
Bone Fracture	12 (2.5%)
Lower limb fracture	5 (1.0%)
Compression fracture	5 (1.0%)
Upper limb fracture	0 (0.0%)
Herpes zoster	5 (1.0%)
Interstitial pneumonia	4 (0.8%)
Tuberculosis	0 (0.0%)
Diabetes mellitus^c^	28 (5.8%)
Cataract^c^	27 (5.6%)
Glaucoma^c^	12 (2.5%)

^a^Data are expressed as mean ± standard deviation

^b^Rapid progressors were defined as those who developed OMDS 5 or above within 2 years from the onset of motor symptoms

^c^These data are based on open-ended questions about comorbidities, Abbreviation: OMDS, Osame motor disability score

Next, we investigated the incidence of comorbidities in the 434 patients (Table 4) who participated in at least one annual interview (Fig. 1b). They had almost the same patient characteristics as the entire HAM-net registry. The more prevalent comorbidities were bone fractures, herpes zoster, and uveitis; the incidence rates were 55.5 (95% CI: 44.0–69.8), 10.4 (95% CI: 6.2–17.4), and 6.5 (95% CI: 3.3–12.7) per 1000 person-years, respectively (Table 4). In more detail, regarding fractures, the incidence rates of lower limb fractures, compression fractures, and upper limb fractures were 22.6, 22.4, and 3.7 per 1000 person-years, respectively. To investigate the impact of baseline OMDS on the incidence of fractures, we calculated the incidence of fractures based on OMDS (OMDS 1–4, OMDS 5, OMDS 6, and OMDS 7–13). As shown in Table 4, the OMDS categories with high incidence were OMDS 6 for all bone fractures, OMDS 7–13 for lower limb fractures, and OMDS 6 for compression fractures. The OMDS category with low incidence was OMDS 1–4 for all types of fractures. The prevalence and incidence of tuberculosis were 0.0 and 0.0 (95% CI: 0.0–2.8) per 1000 person-years, respectively. Adult T-cell leukemia-lymphoma was one of the most significant comorbidities of HAM/TSP, although its prevalence and incidence are not described here because this is the subject of a separate paper currently in preparation.

**Table 4:** Patient characteristics and incidence of comorbidities of HAM-net-registered patients

HAM-net-registered patients observed for at least one year (*n* = 434)	
Sex: Female	327 (75.3%)
Age at baseline (year)^a^	61.9 ± 10.6
Age at onset (year)^a^	44.4 ± 14.8
Disease duration^a^ (time from onset to the baseline)	16.3 ± 11.1
Baseline OMDS^a^	5.7 ± 2.2
Rapid progressors^b^	83 (19.1%)
Comorbidities	Incidence per 1000 person-years^c^
Uveitis	6.5 (3.3–12.7)
Sjogren’s syndrome	0.8 (0.1–4.3)
Rheumatoid arthritis	3.0 (1.2–7.7)
All Bone Fracture	55.5 (44.0–69.8)
OMDS 1–4	35.9 (19.5–66.1)
OMDS 5	57.4 (39.2–84.2)
OMDS 6	73.2 (46.3–115.8)
OMDS 7–13	56.0 (35.4–88.6)
Lower limb fracture	22.6 (15.8–32.3)
OMDS 1–4	3.5 (0.6–19.6)
OMDS 5	23.7 (13.3–42.5)
OMDS 6	24.1 (11.1–52.6)
OMDS 7–13	36.8 (21.0–64.3)
Compression fracture	22.4 (15.7–32.0)
OMDS 1–4	10.6 (3.6–31.1)
OMDS 5	19.3 (10.1–36.6)
OMDS 6	44.0 (24.6–78.9)
OMDS 7–13	20.7 (10.0–42.8)
Upper limb fracture	3.7 (1.6–8.6)
Herpes zoster	10.4 (6.2–17.4)
Interstitial pneumonia	1.5 (0.4–5.4)
Tuberculosis	0.0 (0.0–2.8)
Diabetes mellitus^d^	6.2 (3.1–12.2)
Cataract^d^	9.4 (5.4–16.5)
Glaucoma^d^	2.3 (0.8–6.7)

^a^Data are expressed as mean ± standard deviation

^b^Rapid progressors were defined as those who developed OMDS 5 or above within 2 years from the onset of motor symptoms

^c^Data are expressed as point estimates and 95% confidence intervals

^d^These data are based on open-ended questions about comorbidities, Abbreviation: OMDS, Osame motor disability score

### Drug utilization situation

As shown in Table 5, we examined the treatment experience of patients in the HAM-net registry before the initial interview, at the time of the initial interview, and in each subsequent year. The percent of patients with a history of oral steroid therapy, methylprednisolone pulse therapy, and interferon-α treatment were 67.1, 39.3, and 35.0%, respectively. The percentages of patients who received each therapy at the time of the initial interview were 41.8, 1.4, and 2.9%, respectively. We found that in each subsequent year the percent of patients who received each treatment were 48.2–50.7% (oral steroid therapy), 3.6–7.6% (pulse therapy), and 2.6–3.5% (interferon-α treatment). Next, we examined the continuation rate of each treatment (Additional file 2: Table S1). Of the 123 subjects, 112 (91.1%) continued to take oral steroid therapy for 3 years between the 2nd and the 5th year interviews. In contrast, only 7 of 18 (38.9%) and 7 of 11 (63.6%) continued on steroid pulse therapy and interferon-α treatment, respectively, during the same period. The daily dose of oral steroid prednisolone for treated patients (*n* = 167) at the time of the initial interview was 7.4 ± 5.2 mg/day (mean ± standard deviation), 5.0 mg/day (median) (Additional file 2: Table S2).

**Table 5:** Drug utilization of HAM-net-registered patients

		Before the initial interview (n = 486)	At the time of the initial interview (n = 486)	In a year until the 2nd-year interview (n = 434)	In a year until the 3rd-year interview (*n* = 370)	In a year until the 4th-year interview (*n* = 304)	In a year until the 5th-year interview (*n* = 257)
Oral steroid therapy	(+)	326 (67.1%)	203 (41.8%)	209 (48.2%)	186 (50.3%)	154 (50.7%)	124 (48.2%)
	(−)	134 (27.6%)	257 (52.9%)	214 (49.3%)	182 (49.2%)	148 (48.7%)	130 (50.6%)
	unknown	26 (5.3%)	26 (5.3%)	11 (2.5%)	2 (0.5%)	2 (0.7%)	3 (1.2%)
Methyl- prednisolone pulse therapy	(+)	191 (39.3%)	7 (1.4%)	33 (7.6%)	21 (5.7%)	11 (3.6%)	10 (3.9%)
	(−)	267 (54.9%)	449 (92.4%)	391 (90.1%)	346 (93.5%)	292 (96.1%)	244 (94.9%)
	unknown	28 (5.8%)	30 (6.2%)	10 (2.3%)	3 (0.8%)	1 (0.3%)	3 (1.2%)
Interferon-α treatment	(+)	170 (35.0%)	14 (2.9%)	15 (3.5%)	12 (3.2%)	8 (2.6%)	8 (3.1%)
	(−)	295 (60.7%)	448 (92.2%)	410 (94.5%)	356 (96.2%)	295 (97.0%)	247 (96.1%)
	unknown	21 (4.3%)	24 (4.9%)	9 (2.1%)	2 (0.5%)	1 (0.3%)	2 (0.8%)

### Incidence of steroid-related complications

As shown in Table 6, we investigated the incidence of steroid-related complications by steroid treatment status among the 434 patients that were observed for at least one-year. The incidence of bone fracture tended to be higher in the steroid groups than in the untreated group. The incidences were 48.3 (95% CI: 33.0–70.8) in the untreated group, 61.0 (95% CI: 45.1–82.5) in the steroid group, and 65.6 (95% CI: 47.4–90.8) in the continued steroid group per 1000 person-years. The rates of lower limb fractures in the three groups were 21.8, 24. 2, and 26.7, respectively, per 1000 person-years, and the rates of compression fractures were 16.1, 25.4 and 28.1, respectively, per 1000 person-years.

**Table 6:** Incidence of steroid-related complications by steroid treatment status

	Patients with HAM/TSP without steroid therapy during the observation period (Untreated group, *n* = 185)	Patients with HAM/TSP with at least once steroid therapy during the observation period (Steroid group, *n* = 225)	Patients with HAM/TSP who had continued steroid therapy during the observation period (Continued steroid group, *n* = 181)
OMDS 1–4	46 (24.9%)	51 (22.7%)	39 (21.5%)
OMDS 5	54 (29.2%)	91 (40.4%)	71 (39.2%)
OMDS 6	34 (18.4%)	38 (16.9%)	34 (18.8%)
OMDS 7–13	51 (27.6%)	45 (20.0%)	37 (20.4%)
Steroid-related complications	Incidence per 1000 person-years^a^	Incidence per 1000 person-years^a^	Incidence per 1000 person-years^a^
Bone fracture	48.3 (33.0–70.8)	61.0 (45.1–82.5)	65.6 (47.4–90.8)
OMDS 1–4	42.9 (18.3–100.5)	33.4 (14.2–78.1)	35.0 (13.6–90.1)
OMDS 5	26.2 (10.2–67.3)	76.5 (50.0–116.9)	83.4 (52.8–131.9)
OMDS 6	72.4 (36.7–142.9)	60.3 (29.2–124.5)	48.4 (20.7–113.2)
OMDS 7–13	56.8 (29.9–107.9)	60.9 (32.0–115.7)	77.9 (41.0–148.0)
Lower limb fracture	21.8 (12.5–38.1)	24.2 (15.1–38.7)	26.7 (16.2–44.0)
OMDS 1–4	0.0 (0.0–30.5)	6.6 (1.2–37.6)	8.7 (1.5–49.2)
OMDS 5	25.8 (10.0–66.4)	25.1 (12.1–51.7)	c
OMDS 6	35.6 (13.8–91.4)	8.4 (1.5–47.5)	0.0 (0.0–36.1)
OMDS 7–13	25.4 (9.9–65.4)	51.8 (26.3–102.3)	65.6 (33.2–129.4)
Compression fracture	16.1 (8.5–30.6)	25.4 (16.1–40.2)	28.1 (17.3–45.7)
OMDS 1–4	8.4 (1.5–47.5)	13.1 (3.6–47.7)	17.1 (4.7–62.3)
OMDS 5	0.0 (0.0–24.0)	28.3 (14.4–55.9)	26.8 (12.3–58.5)
OMDS 6	35.5 (13.8–91.4)	42.4 (18.1–99.2)	47.5 (20.3–111.2)
OMDS 7–13	23.8 (9.3–61.2)	19.4 (6.6–57.0)	24.5 (8.3–72.0)
Herpes Zoster	8.8 (3.8–20.6)	12.7 (6.7–24.1)	12.3 (6.0–25.4)
Diabetes mellitus^b^	9.0 (3.9–21.2)	4.4 (1.5–13.1)	5.6 (1.9–16.5)
Cataract^b^	9.2 (3.9–21.5)	9.1 (4.2–19.8)	7.5 (2.9–19.4)
Glaucoma^b^	3.6 (1.0–13.0)	1.4 (0.3–8.2)	1.8 (0.3–10.2)

^a^Data are expressed as point estimates and 95% confidence intervals

^b^These data are based on open-ended questions about complications, Abbreviation: OMDS, Osame motor disability score

The incidence rate of herpes zoster also tended to be higher in the group of patients treated with steroids than in untreated patients. Per 1000 person-years, the rates were 8.8 (95%CI: 3.8–20.6) for untreated patients, 12.7 (95%CI: 6.7–24.1) for treated patients, and 12.3 (95%CI: 6.0–25.4) for continuously treated patients. In contrast, these trends were not observed for diabetes mellitus, cataracts, and glaucoma. Since the incidence of fractures varied based on OMDS (Table 4), we examined the distribution of OMDS in each of the three patient subgroups and found that OMDS tended to be skewed with the percent of OMDS 7–13 being higher in the untreated group than in the steroid treated groups (Table 6). However, the proportion of patients with OMDS ≥5 was almost similar among the three subgroups (75.1, 77.3, and 78.5%).

### Chronological changes in OMDS in the one-year observation group

To assess the chronological changes in OMDS, a new analysis set that excluded patients who met the exclusion/drop-out criteria was used (Fig. 2). The baseline characteristics of the new one-year observation group (Table 7) were nearly identical to those of the entire HAM-net-registered patients. We also found no differences in gender and baseline age across the four subgroups (steroid, steroid-history, untreated, and miscellaneous). However, there was a significant difference in the baseline OMDS between the four subgroups (*p* < 0.001). Specifically, the untreated group had substantially lower baseline OMDSs than those of both the steroid and the steroid-history groups (5.0 vs. 5.7, 6.5). We also found differences in age at onset and disease duration across the four subgroups (*p* = 0.009 and p < 0.001). The steroid group had a higher onset age (47.4 years vs. 40.4 years) and a shorter disease duration (13.9 years vs. 20.6 years), compared to the steroid-history group. Interestingly, there was a tendency that the proportion of rapid progressors was high in the steroid group (26.7%) and low in the untreated group (14.1%).

**Table 7:** Baseline characteristics of HAM-net-registered patients who had been observed for one year (*n* = 346)

	All patients (*n* = 346)	Steroid (S) group (*n* = 131)	Steroid- history (SH) group (n = 82)	Untreated (U) group (*n* = 85)	Miscella- neous (M) group (n = 48)	*p* value	Groups with significant difference ^c)^
Sex: Female	260 (75.1%)	97 (74.0%)	60 (73.2%)	67 (78.8%)	36 (75.0%)	0.832 ^a)^	–
Age at baseline (year)^d^	61.8 ± 10.8	62.1 ± 9.30	62.2 ± 11.7	61.5 ± 11.9	60.7 ± 11.0	0.875 ^b)^	–
Age at onset (year)^d^	44.6 ± 14.8	47.4 ± 13.6	40.4 ± 15.2	45.0 ± 15.0	43.1 ± 16.0	0.009 ^b)^	S > SH
Disease duration^d^ (Time from onset to initial interview)	16.1 ± 11.1	13.9 ± 10.0	20.6 ± 10.8	14.9 ± 11.7	16.7 ± 11.3	< 0.001 ^b)^	SH > S, SH > U
Baseline OMDS^d^	5.7 ± 2.2	5.7 ± 1.9	6.5 ± 2.5	5.0 ± 2.1	5.8 ± 2.2	< 0.001 ^b)^	SH > S > U
Rapid progressors^e^	68 (19.7%)	35 (26.7%)	14 (17.1%)	12 (14.1%)	7 (14.6%)	0.075 ^a)^	–

Statistical methods: ^a)^ By chi-square test, ^b)^ By analysis of variance, ^c)^ By Tukey post hoc tests

^d^Data are expressed as mean ± standard deviation, ^e^ Rapid progressors were defined as those who developed OMDS 5 or above within 2 years from the onset of motor symptoms. Abbrevations: OMDS, Osame motor disability score

Additionally, one-year changes in OMDS in the one-year observation group and its four subgroups were examined (Additional file 2: Tables S3–S7). As shown in Table 8, in the one-year observation group, the difference between baseline OMDS (5.74 ± 2.22) and OMDS at the time of the second survey (5.94 ± 2.29) was 0.20 (95% CI: 0.14–0.25), suggesting that their motor function deteriorated significantly in a year (p < 0.001). In all four subgroups, OMDS deteriorated during the one-year observation period. The magnitudes of the differences were, in decreasing order, the steroid-history group (0.26), steroid group (0.24), untreated group (0.13), and miscellaneous group (0.10). Lastly, we performed the same analyses in an analysis set limited to the patients whose OMDS were 3–6 (*n* = 239, Additional file 1: Figure S1b). This specific analysis set showed the same tendency as the result obtained from the 346 patients (Additional file 2: Tables S8 and S9).

**Table 8:** Changes in Osame motor disability score (OMDS) in patients with HAM/TSP who had been observed for one year (*n* = 346)

	Baseline^a^	2nd-year point^a^	∆OMDS per year^b^	*p* value ^c^
All patients	5.74 ± 2.22	5.94 ± 2.29	0.20 (0.14–0.25)	< 0.001
Steroid group	5.73 ± 1.95	5.97 ± 2.05	0.24 (0.13–0.34)	< 0.001
Steroid-history group	6.54 ± 2.48	6.79 ± 2.61	0.26 (0.13–0.38)	< 0.001
Untreated group	4.95 ± 2.12	5.08 ± 2.16	0.13 (0.06–0.20)	0.001
Miscellaneous group	5.81 ± 2.17	5.92 ± 2.06	0.10 (0.02–0.23)	< 0.001

^a^Data are expressed as mean ± standard deviation

^b^Data are expressed as point estimates and 95% confidence intervals

^c^Statistical methods used the paired t-test

### Chronological changes in OMDS in the four-year observation group

As shown in Table 9, we first examined the baseline characteristics in the four-year observation group (*n* = 148, Fig. 2e) and its four subgroups. These 148 patients had characteristics similar to that of all 486 subjects enrolled in the HAM-net database (Table 3) except for the difference in the percentage of rapid progressors (14.2% vs. 19.8%, respectively). We found no differences in gender, age at baseline, age at onset, disease duration, and the percentage of rapid progressors across the four subgroups (Table 9). However, there was a significant difference in the baseline OMDS (*p* = 0.012).

**Table 9:** Baseline characteristics of patients with HAM/TSP who had been observed for four years (*n* = 148)

	All patients (*n* = 148)	Steroid (S) group (n = 47)	Steroid- history (SH) group (n = 36)	Untreated (U) group (n = 32)	Miscella- neous (M) group (n = 33)	*p* value	Groups with significant difference ^c)^
Sex: Female	112 (75.7%)	36 (76.6%)	25 (69.4%)	24 (75.0%)	27 (81.8%)	0.690 ^a)^	–
Age at baseline (year)^d^	61.8 ± 9.7	63.0 ± 7.5	61.4 ± 9.7	60.6 ± 12.4	61.5 ± 9.7	0.740 ^b)^	–
Age at onset (year)^d^	43.4 ± 14.4	47.5 ± 13.0	40.5 ± 15.2	42.2 ± 13.1	41.7 ± 15.7	0.113 ^b)^	–
Disease duration^d^ (Time from onset to initial interview)	17.5 ± 10.4	15.7 ± 9.4	19.8 ± 10.0	16.6 ± 10.6	18.3 ± 11.9	0.300 ^b)^	–
Baseline OMDS^d^	5.8 ± 2.2	6.0 ± 2.3	6.6 ± 2.4	4.8 ± 1.4	5.7 ± 2.1	0.012 ^b)^	SH > U
Rapid progressors^e^	21 (14.2%)	10 (21.3%)	5 (13.9%)	3 (9.4%)	3 (9.1%)	0.354 ^a)^	–

Statistical methods: ^a)^ By chi-square test, ^b)^ By analysis of variance, ^c)^ By Tukey post hoc tests

^d^Data are expressed as mean ± standard deviation, ^e^ Rapid progressors were defined as those who developed OMDS 5 or above within 2 years from the onset of motor symptoms. Abbreviation: OMDS, Osame motor disability score

We next examined the four-year changes in OMDS in the four-year observation group and its four subgroups (Additional file 2: Tables S10–S14; Additional file 3: Figure S2). As shown in Table 10, when all patients in the four-year observation group were evaluated together, the difference between baseline OMDS (5.80 ± 2.19) and OMDS at the time of the fifth survey (6.37 ± 2.31) was 0.57 (95% CI: 0.42–0.73), indicating that their motor function deteriorated significantly in four years (*p* < 0.001). When each subgroup was compared over time, OMDS declined during the four-year observation period. The magnitudes of the differences were, in decreasing order, the steroids-history group (0.67), steroids (0.64), miscellaneous (0.55), and the untreated group (0.41). Lastly, we performed the same analyses in an analysis set limited to the patients whose OMDS were 3–6 with similar results (Additional file 1: Figure S1e; Additional file 2: Tables S15 and S16).

**Table 10:** Changes in Osame motor disability score (OMDS) in patients with HAM/TSP who had been observed for four years (*n* = 148)

	Baseline^a^	2nd-year point^a^	3rd-year point^a^	4th-year point^a^	5th-year point^a^	∆OMDS per four years^b^	*p* value ^c^
All patients	5.80 ± 2.19	5.99 ± 2.31	6.21 ± 2.36	6.28 ± 2.34	6.37 ± 2.31	0.57 (0.42–0.73)	< 0.001
Steroid group	5.96 ± 2.27	6.15 ± 2.35	6.40 ± 2.48	6.47 ± 2.51	6.60 ± 2.49	0.64 (0.30–0.98)	< 0.001
Steroid-history group	6.56 ± 2.42	6.81 ± 2.64	7.08 ± 2.61	7.14 ± 2.54	7.22 ± 2.50	0.67 (0.32–1.01)	< 0.001
Untreated group	4.84 ± 1.44	5.00 ± 1.63	5.19 ± 1.75	5.22 ± 1.81	5.25 ± 1.70	0.41 (0.13–0.68)	0.005
Miscellaneous group	5.67 ± 2.13	5.85 ± 2.14	5.97 ± 2.08	6.09 ± 1.94	6.21 ± 1.93	0.55 (0.23–0.87)	0.002

^a^Data are expressed as mean ± standard deviation

^b^Data are expressed as point estimates and 95% confidence intervals

^c^Statistical methods used the paired t-test (baseline vs. 5th-year point)

### Changes in OMDS for patients treated with interferon-α

We next examined the effect of interferon-α treatment on OMDS in the analysis set that could be observed for four years (*n* = 148, Fig. 2e) and its three subgroups (Table 11). Only 10 patients had received interferon-α treatment, of which 7 worsened (70.0%), and 3 remained unchanged (30.0%). Five of the seven patients who got worse and two of the three patients who remained unchanged were also receiving steroid therapy at the same time.

**Table 11:** Four-year changes in Osame motor disability score (OMDS) in the three subgroups classified by interferon-α treatment conditions (*n* = 148)

	OMDS change			
	Improved	No change	Worsened	Total
Patients with HAM/TSP without interferon-α treatment during the observation period	2 (1.5%)	86 (65.6%)	43 (32.8%)	131 (100.0%)
Patients with HAM/TSP with at least once interferon-α treatment during the observation period	0 (0.0%)	3 (30.0%)	7 (70.0%)	10 (100.0%)
Patients with HAM/TSP with at least once unknown treatment condition during the observation period	0 (0.0%)	5 (71.4%)	2 (28.6%)	7 (100.0%)
Total	2 (1.4%)	94 (63.5%)	52 (35.1%)	148 (100.0%)

## Discussion

In this study, using “HAM-net” patient registry data, we provided real-world data on chronological changes in OMDS in patients with HAM/TSP according to their treatment regimens. This information has the potential to be used as historical controls. These data show that the lower limb motor function in patients with HAM/TSP significantly deteriorates every year with or without treatment. Indeed, when we analyzed patient groups suitable for the evaluation of OMDS, after excluding patients with factors affecting the lower limb motor function, the mean change in OMDS was + 0.20 (95%CI: 0.14–0.25) over a year in the one-year observation group and it was + 0.57 (95%CI: 0.42–0.73) at four years in the four-year observation group (Tables 8 and 10). For both the one-year and four-year observation groups, OMDS was significantly worse over time in all four subgroups (steroid, steroid-history, untreated, and miscellaneous).

These results indicate the limitations of steroid therapy and strongly suggest the need for new treatments. As an example of the expected effect of new therapeutic agents, the capability of preventing OMDS deterioration or improving OMDS can be considered. Based on the data (+ 0.20/year) obtained from the one-year observation group this time, it takes five years for OMDS to deteriorate by one grade. Also, in the retrospective data previously collected from HAM-net, we found that it took 4 years to deteriorate from OMDS 4 to 5 and 4.5 years to worsen from OMDS 5 to 6 [9]. Taking these points into consideration, a one-grade improvement in OMDS that could be produced by a new treatment would mean that the condition of patients with HAM/TSP had improved back to the level they enjoyed 4 to 5 years ago. Therefore, an improvement in the OMDS metric is clinically significant.

This study also identified three important points about steroid treatment that had been previously unknown. First, our data suggest that steroid therapy is considered an effective intervention for HAM/TSP by many physicians and patients. Even though the authorities in Japan have not approved steroid therapy for HAM/TSP, it is actually administered to approximately half of HAM-net-registered patients (Table 5), and the continuation rate has exceeded 90% (Additional file 2: Table S1). On the other hand, although the relevant authorities in Japan have approved the interferon-α treatment for HAM/TSP, the number of patients for whom it has been administered is as small as approximately 3% (Table 5), and we found that OMDS for patients who received this treatment often worsened (Table 11). This result suggests that interferon-α is not being used very aggressively; moreover, when used, it has no long-term ameliorating effects on patients with progressive disease.

Second, the present study suggests that while HAM/TSP patients with high disease activity, including the rapid progressors, were being actively treated with steroids, HAM/TSP patients with low disease activity were not receiving such treatment. Actually, there was a higher percentage of rapid progressors in the steroid group than in the untreated group (Tables 7 and 9). Also, relative to patients in the steroid group, those in the untreated group tended to progress more slowly (Table 8, change in OMDS in one-year observation group: 0.13 vs. 0.24; Table 10, change in OMDS in four-year observation group: 0.41 vs. 0.64).

So far, we have shown that the disease activity of patients with HAM/TSP is not uniform and that their levels can be distinctly classified; the long-term prognosis for patients with high disease activity is considerably worse compared with that of patients with low disease activity [10]. For these reasons, we believe that so-called “stratified treatment” is essential, such that the disease activity is evaluated before the start of treatment, and the treatment course is decided accordingly. The results of this study reflect that this specific approach has already been performed empirically at the point-of-care. Moreover, it has already been reported that continuous low-dose oral prednisolone improves the relatively long-term prognosis in patients with HAM/TSP [12]. Considering this effectiveness, the symptoms of the steroid group patients may have been much more advanced if not treated with steroids.

Third, this investigation suggests that steroid therapy for HAM/TSP patients increases the incidence of steroid-related complications (bone fractures and herpes zoster), even at low doses (median daily dose of prednisolone 5 mg, Additional file 2: Table S2). With regard to the steroid-related complications in patients with HAM/TSP, there have been some reports of the frequency of side effects that have occurred while using relatively high doses of prednisolone (0.5–1.0 mg/kg/day) for several months [13, 14]. However, there are no reports of the incidence of side effects that have occurred while using low doses of prednisolone for several years to treat patients with HAM/TSP. In our study, patient groups treated with steroids tended to have higher rates of fractures, regardless of the fracture types (Table 6). The proportion of patients with OMDS ≥5 was almost similar between the untreated and steroid treated subgroups (Table 6 upside); therefore, OMDS may not be a confounding factor that increases fractures, and it is likely that steroids increase the incidence of fractures. In this study, the rate of bisphosphonate use in patients receiving steroid treatment was unknown. Future research should investigate this point and confirm whether sufficient preventive measures have been taken.

The current study also revealed the prevalence of several comorbidities that are related to HAM/TSP (Table 3). Most noticeably, the prevalence of uveitis, Sjogren’s syndrome, and rheumatoid arthritis was high. These diseases have also been reported as the frequently observed complications of HAM/TSP in Kagoshima, a HTLV-1-endemic area in Japan [15]. In our study, the prevalence of uveitis in HAM-net-registered patients was 7.6%. According to the results of an ophthalmologic survey of patients with HAM/TSP in Salvador, Brazil, the prevalence of uveitis was 2% [16]. In the Kagoshima study, uveitis was found in 4% of the patients with HAM/TSP [15]. Since the prevalence of HTLV-1 uveitis in HTLV-1 carriers is around 0.1% [17], the prevalence of uveitis in patients with HAM/TSP is high.

The prevalence of Sjogren’s syndrome in Japan has been reported to be 0.05% [18], but the rate for patients with HAM/TSP in this study was remarkably higher at 3.7% (Table 3), suggesting that patients with HAM/TSP apparently have a higher prevalence of Sjogren’s syndrome than the general population. This observation is consistent with another previous finding that there are many patients with HAM/TSP who have Sjogren’s syndrome in Nagasaki [19] and in Kagoshima [15], the HTLV-1-endemic areas of Japan, suggesting a relationship between the pathogenesis of both diseases. Likewise, the prevalence of rheumatoid arthritis in Japan is 0.6–1.0% [20], but the rate in patients with HAM/TSP in this study was 2.7% (Table 3). Therefore, patients with HAM/TSP may also have a higher prevalence of rheumatoid arthritis than the general population. This finding is consistent with other previous reports that there are more HTLV-1-infected individuals among RA patients [21] and that HTLV-1 carriers have a higher prevalence of RA than non-infected individuals [22].

This study also revealed the incidence of several comorbidities in patients with HAM/TSP (Table 4). To our knowledge, no previous study has reported the incidence of comorbidities in patients with HAM/TSP. The incidence of herpes zoster in HAM-net-registered patients was 10.4 per 1000 person-years (Table 4), while the rate of herpes zoster in people in their 60’s in Miyazaki, a prefecture of Japan on the island of Kyushu, has been reported to be approximately 7 per 1000 person-years [23]. A simple comparison shows a 1.5 times increase in incidence, suggesting a decrease in cellular immunity due to HTLV-1 infection and steroid therapy. However, the onset of tuberculosis was not observed in HAM-net-registered patients.

There are three limitations in this study. One, the analysis is partly based on retrospective data such as onset age and treatment history. Two, OMDS which was used to evaluate motor function is not widely used across the world. Three, it is not possible to statistically evaluate the efficacy of steroid therapy by adjusting patient backgrounds using propensity scores or multivariate analyses while the backgrounds between steroid group and untreated group are different. This issue exists because 41.8% of our patients had already received steroid therapy at the time of the initial interview, and only 17 patients started steroid therapy among those remaining patients who had not previously received it. Therefore, the effectiveness of steroid therapy cannot be determined from this study. In that sense, our previous multicenter retrospective cohort study is important because the efficacy of prednisolone was shown in patients who newly started steroid therapy by comparison with the untreated group [12]. However, as mentioned above, steroid therapy has been applied to patients with high disease activity and has not been able to prevent the deterioration of HAM/TSP in the chronic phase. Thus, the effectiveness of steroids is not sufficient, and there is a great need to develop new treatments.

## Conclusions

The present study revealed the epidemiological information of HAM/TSP that has not been reported until now, such as the incidence of comorbidities and the history of drug utilization. In addition, this study has provided real-world data on chronological changes in lower limb motor dysfunction of patients with HAM/TSP which can now be used as historical controls.

## Supplementary information

**Additional file 1: Figure S1.** Flowchart for showing analysis sets limited to OMDS 3–6. Patients with OMDS 3–6 were extracted from analysis set 2 for sub-analysis, assuming motor function evaluation in clinical trials.

**Additional file 2: Table S1**. Continuation rates of treatments in HAM-net-registered patients. **Table S2**. Distribution of the daily dose of prednisolone at the time of initial interview in HAM-net-registered patients. **Table S3.** Cross-tabulation of OMDS at the time of initial interview versus OMDS at the time of 2nd-year interview (one-year observation group, *n* = 346). **Table S4.** Cross-tabulation of OMDS at the time of initial interview versus OMDS at the time of 2nd-year interview (steroid group, *n* = 131). **Table S5.** Cross-tabulation of OMDS at the time of initial interview versus OMDS at the time of 2nd-year interview (steroid-history group, *n* = 82). **Table S6.** Cross-tabulation of OMDS at the time of initial interview versus OMDS at the time of 2nd-year interview (untreated group, *n* = 85). **Table S7.** Cross-tabulation of OMDS at the time of initial interview versus OMDS at the time of 2nd-year interview (miscellaneous group, *n* = 48). **Table S8.** Baseline characteristics of patients with HAM/TSP with OMDS 3–6 who had been observed for one year (*n* = 239). **Table S9.** Changes in OMDS in patients with HAM/TSP with OMDS 3–6 who had been observed for one year (n = 239). **Table S10.** Cross-tabulation of OMDS at the time of initial interview versus OMDS at the time of 5th-year interview (Four-year observation group, *n* = 148). **Table S11.** Cross-tabulation of OMDS at the time of initial interview versus OMDS at the time of 5th-year interview (steroid group, *n* = 47). **Table S12.** Cross-tabulation of OMDS at the time of initial interview versus OMDS at the time of 5th-year interview (steroid-history group, *n* = 36). **Table S13.** Cross-tabulation of OMDS at the time of initial interview versus OMDS at the time of 5th-year interview (untreated group, *n* = 32). **Table S14.** Cross-tabulation of OMDS at the time of initial interview versus OMDS at the time of 5th-year interview (miscellaneous group, *n* = 33). **Table S15.** Baseline characteristics of patients with HAM/TSP with OMDS 3–6 who had been observed for four years (*n* = 100). **Table S16.** Changes in OMDS in patients with HAM/TSP with OMDS 3–6 who had been observed for four years (n = 100).

**Additional file 3: Figure S2.** Chronological change in OMDS in the four-year observation group (n = 148). Each bar in this bar chart represents 148 patients (four-year observation group, Fig. 2e) as 100% and indicates the percentage of patients belonging to each OMDS at each survey time.

## Data Availability

Most data generated or analyzed during this study are included in this published article [and its supplementary information files]. The other datasets used and/or analyzed during the current study are available from the corresponding author on reasonable request.

## Abbreviations

CI: Confidence intervals
HAM/TSP: HTLV-1-associated myelopathy/tropical spastic paraparesis
HTLV-1: Human T-cell leukemia virus type 1
OMDS: Osame motor disability score
