# Supplementary material for: Real-world clinical course of HTLV-1-associated myelopathy/tropical spastic paraparesis (HAM/TSP) in Japan
Source: Orphanet J Rare Dis. 2019 Oct 21;14:227. doi: 10.1186/s13023-019-1212-4 (PMC6802124; doi:10.1186/s13023-019-1212-4)
Supplement: Supplementary file 1 — Additional file 1: Figure S1. Flowchart for showing analysis sets limited to OMDS 3–6. Patients with OMDS 3–6 were extracted from analysis set 2 for sub-analysis, assuming motor function evaluation in clinical trials. [file 13023_2019_1212_MOESM1_ESM.docx]

**Additional file 1**

Figure S1. Flowchart for showing analysis sets limited to OMDS 3–6

**
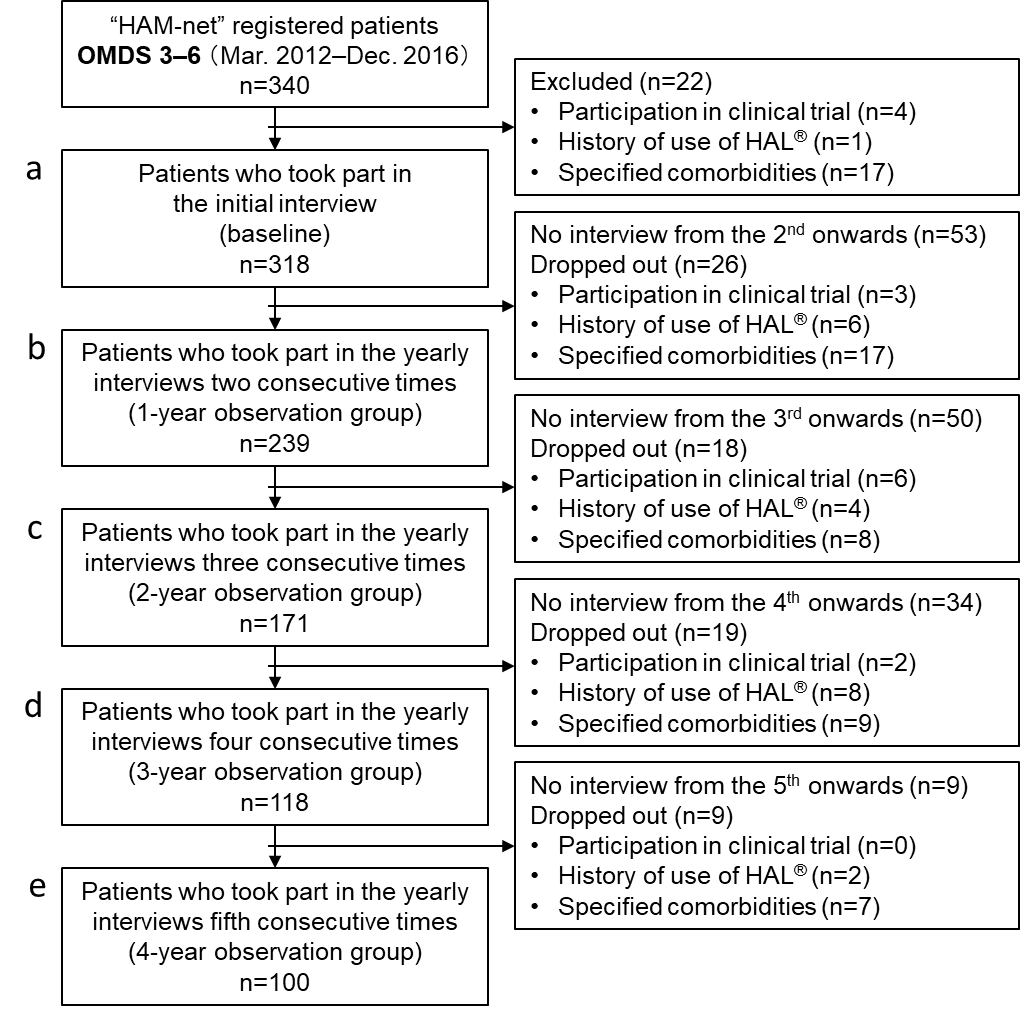
**Patients with OMDS 3–6 were extracted from analysis set 2 for sub-analysis, assuming motor function evaluation in clinical trials. The right half of this chart shows the number of patients who were excluded at each step and the reasons. In this paper, we used only the four-year observation group (n = 100, e) for this sub-analysis.
