# Supplementary material for: Real-world clinical course of HTLV-1-associated myelopathy/tropical spastic paraparesis (HAM/TSP) in Japan
Source: Orphanet J Rare Dis. 2019 Oct 21;14:227. doi: 10.1186/s13023-019-1212-4 (PMC6802124; doi:10.1186/s13023-019-1212-4)
Supplement: Supplementary file 2 — Additional file 2: Table S1. Continuation rates of treatments in HAM-net-registered patients. Table S2. Distribution of the daily dose of prednisolone at the time of initial interview in HAM-net-registered patients. Table S3. Cross-tabulation of OMDS at the time of initial interview versus OMDS at the time of 2nd-year interview (one-year observation group, n = 346). Table S4. Cross-tabulation of OMDS at the time of initial interview versus OMDS at the time of 2nd-year interview (steroid group, n = 131). Table S5. Cross-tabulation of OMDS at the time of initial interview versus OMDS at the time of 2nd-year interview (steroid-history group, n = 82). Table S6. Cross-tabulation of OMDS at the time of initial interview versus OMDS at the time of 2nd-year interview (untreated group, n = 85). Table S7. Cross-tabulation of OMDS at the time of initial interview versus OMDS at the time of 2nd-year interview (miscellaneous group, n = 48). Table S8. Baseline characteristics of patients with HAM/TSP with OMDS 3–6 who had been observed for one year (n = 239). Table S9. Changes in OMDS in patients with HAM/TSP with OMDS 3–6 who had been observed for one year (n = 239). Table S10. Cross-tabulation of OMDS at the time of initial interview versus OMDS at the time of 5th-year interview (Four-year observation group, n = 148). Table S11. Cross-tabulation of OMDS at the time of initial interview versus OMDS at the time of 5th-year interview (steroid group, n = 47). Table S12. Cross-tabulation of OMDS at the time of initial interview versus OMDS at the time of 5th-year interview (steroid-history group, n = 36). Table S13. Cross-tabulation of OMDS at the time of initial interview versus OMDS at the time of 5th-year interview (untreated group, n = 32). Table S14. Cross-tabulation of OMDS at the time of initial interview versus OMDS at the time of 5th-year interview (miscellaneous group, n = 33). Table S15. Baseline characteristics of patients with HAM/TSP with OMDS 3–6 who had been observed [file 13023_2019_1212_MOESM2_ESM.docx]

**Additional file 2**

Table S1. Continuation rates of treatments in HAM-net-registered patients

|  |  | At the time of the 2^nd^-year interview | In a year until the 3^rd^-year interview | In a year until the 4^th^-year interview | In a year until the 5^th^-year interview |
| --- | --- | --- | --- | --- | --- |
| Oral steroid therapy | (+) | 123 | 120 | 117 | 112 |
|  | Continuation rate | 100% | 97.6% | 95.1% | 91.1% |
| Methyl-  prednisolone pulse therapy | (+) | 18 | 12 | 8 | 7 |
|  | Continuation rate | 100% | 66.7% | 44.4% | 38.9% |
| Interferon-α treatment | (+) | 11 | 10 | 8 | 7 |
|  | Continuation rate | 100% | 90.9% | 72.7% | 63.6% |

Table S2. Distribution of the daily dose of prednisolone at the time of initial interview in HAM-net-registered patients

| Daily dose of prednisolone^*^ | n | % |
| --- | --- | --- |
| <5 mg | 42 | 25.1% |
| 5 mg | 51 | 30.5% |
| >5 mg, <10 mg | 15 | 9.0% |
| 10 mg | 40 | 24.0% |
| >10 mg, <30 mg | 19 | 11.4% |
| Total | 167^**^ | 100.0% |

*When taking steroids other than prednisolone, they were converted into the equivalent amount of prednisolone. In addition, in the case of alternate day administration, the dose was multiplied by 0.5 to be regarded as a daily dose.

**Of the 203 patients who were receiving steroid therapy at the time of the initial interview, 36 patients for whom drug name and oral dose were unknown were excluded from the study.

Table S3. Cross-tabulation of OMDS at the time of initial interview versus OMDS at the time of 2^nd^-year interview (one-year observation group, n = 346)

|  | | OMDS at the 2^nd^-year point | | | | | | | | | | | | | | Total |
| --- | --- | --- | --- | --- | --- | --- | --- | --- | --- | --- | --- | --- | --- | --- | --- | --- |
|  |  | 0 | 1 | 2 | 3 | 4 | 5 | 6 | 7 | 8 | 9 | 10 | 11 | 12 | 13 |  |
| Baseline OMDS | 0 | 2 |  |  |  |  |  |  |  |  |  |  |  |  |  | 2 |
|  | 1 |  | 4 |  |  |  |  |  |  |  |  |  |  |  |  | 4 |
|  | 2 |  |  | 9 | 3 | 1 |  |  |  |  |  |  |  |  |  | 13 |
|  | 3 |  |  |  | 9 | 1 |  |  |  |  |  |  |  |  |  | 10 |
|  | 4 |  |  |  |  | 42 | 5 | 2 |  |  |  |  |  |  |  | 49 |
|  | 5 |  |  |  |  | 1 | 106 | 14 | 1 |  |  |  |  |  |  | 122 |
|  | 6 |  |  |  |  |  |  | 47 | 8 | 3 |  |  |  |  |  | 58 |
|  | 7 |  |  |  |  |  |  |  | 17 | 5 | 1 | 1 |  |  |  | 24 |
|  | 8 |  |  |  |  |  |  |  |  | 16 | 3 | 2 |  |  |  | 21 |
|  | 9 |  |  |  |  |  |  |  |  | 1 | 13 | 3 |  |  |  | 17 |
|  | 10 |  |  |  |  |  |  |  |  |  |  | 14 |  |  | 1 | 15 |
|  | 11 |  |  |  |  |  |  |  |  |  |  |  | 2 |  | 1 | 3 |
|  | 12 |  |  |  |  |  |  |  |  |  |  |  |  | 3 |  | 3 |
|  | 13 |  |  |  |  |  |  |  |  |  |  |  |  |  | 5 | 5 |
| Total | | 2 | 4 | 9 | 12 | 45 | 111 | 63 | 26 | 25 | 17 | 20 | 2 | 3 | 7 | 346 |

Table S4. Cross-tabulation of OMDS at the time of initial interview versus OMDS at the time of 2^nd^-year interview (steroid group, n = 131)

|  | | OMDS at the 2^nd^-year point | | | | | | | | | | | | | | Total |
| --- | --- | --- | --- | --- | --- | --- | --- | --- | --- | --- | --- | --- | --- | --- | --- | --- |
|  |  | 0 | 1 | 2 | 3 | 4 | 5 | 6 | 7 | 8 | 9 | 10 | 11 | 12 | 13 |  |
| Baseline OMDS | 0 |  |  |  |  |  |  |  |  |  |  |  |  |  |  |  |
|  | 1 |  |  |  |  |  |  |  |  |  |  |  |  |  |  |  |
|  | 2 |  |  | 4 |  | 1 |  |  |  |  |  |  |  |  |  | 5 |
|  | 3 |  |  |  |  | 1 |  |  |  |  |  |  |  |  |  | 1 |
|  | 4 |  |  |  |  | 18 | 1 | 1 |  |  |  |  |  |  |  | 20 |
|  | 5 |  |  |  |  | 1 | 41 | 7 | 1 |  |  |  |  |  |  | 50 |
|  | 6 |  |  |  |  |  |  | 19 | 3 | 2 |  |  |  |  |  | 24 |
|  | 7 |  |  |  |  |  |  |  | 8 | 1 | 1 | 1 |  |  |  | 11 |
|  | 8 |  |  |  |  |  |  |  |  | 7 |  | 1 |  |  |  | 8 |
|  | 9 |  |  |  |  |  |  |  |  |  | 5 |  |  |  |  | 5 |
|  | 10 |  |  |  |  |  |  |  |  |  |  | 3 |  |  |  | 3 |
|  | 11 |  |  |  |  |  |  |  |  |  |  |  | 1 |  | 1 | 2 |
|  | 12 |  |  |  |  |  |  |  |  |  |  |  |  |  |  |  |
|  | 13 |  |  |  |  |  |  |  |  |  |  |  |  |  | 2 | 2 |
| Total | |  |  | 4 |  | 21 | 42 | 27 | 12 | 10 | 6 | 5 | 1 |  | 3 | 131 |

Table S5. Cross-tabulation of OMDS at the time of initial interview versus OMDS at the time of 2^nd^-year interview (steroid-history group, n = 82)

|  | | OMDS at the 2^nd^-year point | | | | | | | | | | | | | | Total |
| --- | --- | --- | --- | --- | --- | --- | --- | --- | --- | --- | --- | --- | --- | --- | --- | --- |
|  |  | 0 | 1 | 2 | 3 | 4 | 5 | 6 | 7 | 8 | 9 | 10 | 11 | 12 | 13 |  |
| Baseline OMDS | 0 | 2 |  |  |  |  |  |  |  |  |  |  |  |  |  | 2 |
|  | 1 |  | 1 |  |  |  |  |  |  |  |  |  |  |  |  | 1 |
|  | 2 |  |  |  |  |  |  |  |  |  |  |  |  |  |  |  |
|  | 3 |  |  |  |  |  |  |  |  |  |  |  |  |  |  |  |
|  | 4 |  |  |  |  | 7 | 2 |  |  |  |  |  |  |  |  | 9 |
|  | 5 |  |  |  |  |  | 15 | 2 |  |  |  |  |  |  |  | 17 |
|  | 6 |  |  |  |  |  |  | 18 | 1 | 1 |  |  |  |  |  | 20 |
|  | 7 |  |  |  |  |  |  |  | 4 | 4 |  |  |  |  |  | 8 |
|  | 8 |  |  |  |  |  |  |  |  | 3 | 3 | 1 |  |  |  | 7 |
|  | 9 |  |  |  |  |  |  |  |  |  | 5 | 2 |  |  |  | 7 |
|  | 10 |  |  |  |  |  |  |  |  |  |  | 6 |  |  | 1 | 7 |
|  | 11 |  |  |  |  |  |  |  |  |  |  |  | 1 |  |  | 1 |
|  | 12 |  |  |  |  |  |  |  |  |  |  |  |  | 1 |  | 1 |
|  | 13 |  |  |  |  |  |  |  |  |  |  |  |  |  | 2 | 2 |
| Total | | 2 | 1 |  |  | 7 | 17 | 20 | 5 | 8 | 8 | 9 | 1 | 1 | 3 | 82 |

Table S6. Cross-tabulation of OMDS at the time of initial interview versus OMDS at the time of 2^nd^-year interview (untreated group, n = 85)

|  | | OMDS at the 2^nd^-year point | | | | | | | | | | | | | | Total |
| --- | --- | --- | --- | --- | --- | --- | --- | --- | --- | --- | --- | --- | --- | --- | --- | --- |
|  |  | 0 | 1 | 2 | 3 | 4 | 5 | 6 | 7 | 8 | 9 | 10 | 11 | 12 | 13 |  |
| Baseline OMDS | 0 |  |  |  |  |  |  |  |  |  |  |  |  |  |  |  |
|  | 1 |  | 3 |  |  |  |  |  |  |  |  |  |  |  |  | 3 |
|  | 2 |  |  | 5 | 1 |  |  |  |  |  |  |  |  |  |  | 6 |
|  | 3 |  |  |  | 7 |  |  |  |  |  |  |  |  |  |  | 7 |
|  | 4 |  |  |  |  | 11 | 2 |  |  |  |  |  |  |  |  | 13 |
|  | 5 |  |  |  |  |  | 34 | 4 |  |  |  |  |  |  |  | 38 |
|  | 6 |  |  |  |  |  |  | 5 | 3 |  |  |  |  |  |  | 8 |
|  | 7 |  |  |  |  |  |  |  | 1 |  |  |  |  |  |  | 1 |
|  | 8 |  |  |  |  |  |  |  |  | 2 |  |  |  |  |  | 2 |
|  | 9 |  |  |  |  |  |  |  |  |  | 1 | 1 |  |  |  | 2 |
|  | 10 |  |  |  |  |  |  |  |  |  |  | 3 |  |  |  | 3 |
|  | 11 |  |  |  |  |  |  |  |  |  |  |  |  |  |  |  |
|  | 12 |  |  |  |  |  |  |  |  |  |  |  |  | 2 |  | 2 |
|  | 13 |  |  |  |  |  |  |  |  |  |  |  |  |  |  |  |
| Total | |  | 3 | 5 | 8 | 11 | 36 | 9 | 4 | 2 | 1 | 4 |  | 2 |  | 85 |

Table S7. Cross-tabulation of OMDS at the time of initial interview versus OMDS at the time of

2^nd^-year interview (miscellaneous group, n = 48)

|  | | OMDS at the 2^nd^-year point | | | | | | | | | | | | | | Total |
| --- | --- | --- | --- | --- | --- | --- | --- | --- | --- | --- | --- | --- | --- | --- | --- | --- |
|  |  | 0 | 1 | 2 | 3 | 4 | 5 | 6 | 7 | 8 | 9 | 10 | 11 | 12 | 13 |  |
| Baseline OMDS | 0 |  |  |  |  |  |  |  |  |  |  |  |  |  |  |  |
|  | 1 |  |  |  |  |  |  |  |  |  |  |  |  |  |  |  |
|  | 2 |  |  |  | 2 |  |  |  |  |  |  |  |  |  |  | 2 |
|  | 3 |  |  |  | 2 |  |  |  |  |  |  |  |  |  |  | 2 |
|  | 4 |  |  |  |  | 6 |  | 1 |  |  |  |  |  |  |  | 7 |
|  | 5 |  |  |  |  |  | 16 | 1 |  |  |  |  |  |  |  | 17 |
|  | 6 |  |  |  |  |  |  | 5 | 1 |  |  |  |  |  |  | 6 |
|  | 7 |  |  |  |  |  |  |  | 4 |  |  |  |  |  |  | 4 |
|  | 8 |  |  |  |  |  |  |  |  | 4 |  |  |  |  |  | 4 |
|  | 9 |  |  |  |  |  |  |  |  | 1 | 2 |  |  |  |  | 3 |
|  | 10 |  |  |  |  |  |  |  |  |  |  | 2 |  |  |  | 2 |
|  | 11 |  |  |  |  |  |  |  |  |  |  |  |  |  |  |  |
|  | 12 |  |  |  |  |  |  |  |  |  |  |  |  |  |  |  |
|  | 13 |  |  |  |  |  |  |  |  |  |  |  |  |  | 1 | 1 |
| Total | |  |  |  | 4 | 6 | 16 | 7 | 5 | 5 | 2 | 2 |  |  | 1 | 48 |

Table S8. Baseline characteristics of patients with HAM/TSP with OMDS 3–6 who had been observed for one year (n = 239)

|  | All patients  (n = 239) | Steroid (**S**) group  (n = 95) | Steroid-  history (**SH**) group  (n = 46) | Untreated (**U**) group (n = 66) | Miscella-  neous (**M**) group  (n = 32) | *p* value | Groups with significant difference ^c)^ |
| --- | --- | --- | --- | --- | --- | --- | --- |
| Sex: Female | 176 (73.6%) | 72  (75.8%) | 31  (67.4%) | 51  (77.3%) | 22  (68.8%) | 0.574 ^a)^ | — |
| Age at baseline (year)^*^ | 61.4 ± 11.0 | 62.0 ± 9.2 | 61.5 ± 12.5 | 61.3 ± 12.1 | 59.5 ± 11.6 | 0.736 ^b)^ | — |
| Age at onset (year)^*^ | 45.5 ± 14.5 | 48.1 ± 13.3 | 42.3 ± 16.0 | 44.8 ± 14.9 | 43.6 ± 14.7 | 0.113 ^b)^ | — |
| Disease duration^*^ (Time from onset to initial interview) | 14.9 ± 10.7 | 13.1 ± 9.8 | 18.0 ± 10.5 | 15.4 ± 11.5 | 15.1 ± 10.9 | 0.076 ^b)^ | — |
| Baseline OMDS^*^ | 5.0 ± 0.8 | 5.0 ± 0.7 | 5.2 ± 0.8 | 4.7 ± 0.8 | 4.8 ± 0.8 | 0.003 ^b)^ | **SH > U** |
| Rapid progressors^**^ | 45 (18.8%) | 21 (22.1%) | 11 (23.9%) | 8 (12.1%) | 5 (15.6%) | 0.308 ^a)^ | — |

Statistical methods: a) By chi-square test, b) By analysis of variance, c) By Tukey post hoc tests

*Data are expressed as mean ± standard deviation, ** Rapid progressors were defined as those who developed OMDS 5 or above within 2 years from the onset of motor symptoms. OMDS, Osame motor disability score

Table S9. Changes in OMDS in patients with HAM/TSP with OMDS 3–6 who had been observed for one year (n = 239)

|  | Baseline* | 2^nd^-year point^*^ | ∆OMDS per year** | *p* value ^†^ |
| --- | --- | --- | --- | --- |
| All patients | 4.95 ± 0.78 | 5.12 ± 0.92 | 0.16 (0.11–0.22) | <0.001 |
| Steroid group | 5.02 ± 0.71 | 5.22 ± 0.90 | 0.20 (0.09–0.31) | <0.001 |
| Steroid-history group | 5.24 ± 0.77 | 5.39 ± 0.86 | 0.15 (0.03–0.28) | 0.018 |
| Untreated group | 4.71 ± 0.82 | 4.85 ± 0.95 | 0.14 (0.05–0.22) | 0.002 |
| Miscellaneous group | 4.84 ± 0.81 | 4.97 ± 0.90 | 0.13 (0.03–0.28) | 0.103 |

*Data are expressed as mean ± standard deviation, **Data are expressed as point estimates and 95% confidence intervals, † Statistical methods used a paired t-test

Table S10. Cross-tabulation of OMDS at the time of initial interview versus OMDS at the time of 5^th^-year interview (Four-year observation group, n = 148)

|  | | OMDS at the 5^th^-year point | | | | | | | | | | | | | | Total |
| --- | --- | --- | --- | --- | --- | --- | --- | --- | --- | --- | --- | --- | --- | --- | --- | --- |
|  |  | 0 | 1 | 2 | 3 | 4 | 5 | 6 | 7 | 8 | 9 | 10 | 11 | 12 | 13 |  |
| Baseline OMDS | 0 |  |  |  |  | 1 |  |  |  |  |  |  |  |  |  | 1 |
|  | 1 |  | 1 |  |  |  |  |  |  |  |  |  |  |  |  | 1 |
|  | 2 |  |  | 3 |  | 2 | 1 |  |  |  |  |  |  |  |  | 6 |
|  | 3 |  |  |  | 1 | 2 | 1 |  |  |  |  |  |  |  |  | 4 |
|  | 4 |  |  |  |  | 17 | 5 | 1 |  |  |  |  |  |  |  | 23 |
|  | 5 |  |  |  |  |  | 32 | 6 | 5 | 1 |  |  |  |  |  | 44 |
|  | 6 |  |  |  |  |  |  | 16 | 5 | 5 | 2 | 1 |  |  |  | 29 |
|  | 7 |  |  |  |  |  | 1 | 1 | 5 | 2 | 1 | 2 |  |  |  | 12 |
|  | 8 |  |  |  |  |  |  |  |  | 6 | 1 |  |  |  |  | 7 |
|  | 9 |  |  |  |  |  |  |  |  |  | 5 | 5 |  |  |  | 10 |
|  | 10 |  |  |  |  |  |  |  |  |  |  | 5 | 1 | 1 | 1 | 8 |
|  | 11 |  |  |  |  |  |  |  |  |  |  |  | 1 |  |  | 1 |
|  | 12 |  |  |  |  |  |  |  |  |  |  |  |  |  |  |  |
|  | 13 |  |  |  |  |  |  |  |  |  |  |  |  |  | 2 | 2 |
| Total | |  | 1 | 3 | 1 | 22 | 40 | 24 | 15 | 14 | 9 | 13 | 2 | 1 | 3 | 148 |

Table S11. Cross-tabulation of OMDS at the time of initial interview versus OMDS at the time of 5^th^-year interview (steroid group, n = 47)

|  | | OMDS at the 5^th^-year point | | | | | | | | | | | | | | Total |
| --- | --- | --- | --- | --- | --- | --- | --- | --- | --- | --- | --- | --- | --- | --- | --- | --- |
|  |  | 0 | 1 | 2 | 3 | 4 | 5 | 6 | 7 | 8 | 9 | 10 | 11 | 12 | 13 |  |
| Baseline OMDS | 0 |  |  |  |  |  |  |  |  |  |  |  |  |  |  |  |
|  | 1 |  |  |  |  |  |  |  |  |  |  |  |  |  |  |  |
|  | 2 |  |  | 1 |  | 1 |  |  |  |  |  |  |  |  |  | 2 |
|  | 3 |  |  |  |  |  |  |  |  |  |  |  |  |  |  |  |
|  | 4 |  |  |  |  | 9 | 1 |  |  |  |  |  |  |  |  | 10 |
|  | 5 |  |  |  |  |  | 7 | 2 | 3 | 1 |  |  |  |  |  | 13 |
|  | 6 |  |  |  |  |  |  | 4 | 1 | 2 | 1 | 1 |  |  |  | 9 |
|  | 7 |  |  |  |  |  | 1 | 1 |  |  |  | 1 |  |  |  | 3 |
|  | 8 |  |  |  |  |  |  |  |  | 2 |  |  |  |  |  | 2 |
|  | 9 |  |  |  |  |  |  |  |  |  | 1 | 3 |  |  |  | 4 |
|  | 10 |  |  |  |  |  |  |  |  |  |  | 1 | 1 |  |  | 2 |
|  | 11 |  |  |  |  |  |  |  |  |  |  |  | 1 |  |  | 1 |
|  | 12 |  |  |  |  |  |  |  |  |  |  |  |  |  |  |  |
|  | 13 |  |  |  |  |  |  |  |  |  |  |  |  |  | 1 | 1 |
| Total | |  |  | 1 |  | 10 | 9 | 7 | 4 | 5 | 2 | 6 | 2 |  | 1 | 47 |

Table S12. Cross-tabulation of OMDS at the time of initial interview versus OMDS at the time of 5^th^-year interview (steroid-history group, n = 36)

|  | | OMDS at the 5^th^-year point | | | | | | | | | | | | | | Total |
| --- | --- | --- | --- | --- | --- | --- | --- | --- | --- | --- | --- | --- | --- | --- | --- | --- |
|  |  | 0 | 1 | 2 | 3 | 4 | 5 | 6 | 7 | 8 | 9 | 10 | 11 | 12 | 13 |  |
| Baseline OMDS | 0 |  |  |  |  | 1 |  |  |  |  |  |  |  |  |  | 1 |
|  | 1 |  | 1 |  |  |  |  |  |  |  |  |  |  |  |  | 1 |
|  | 2 |  |  |  |  |  |  |  |  |  |  |  |  |  |  |  |
|  | 3 |  |  |  |  |  |  |  |  |  |  |  |  |  |  |  |
|  | 4 |  |  |  |  | 2 | 1 |  |  |  |  |  |  |  |  | 3 |
|  | 5 |  |  |  |  |  | 4 | 1 | 1 |  |  |  |  |  |  | 6 |
|  | 6 |  |  |  |  |  |  | 6 | 1 | 2 |  |  |  |  |  | 9 |
|  | 7 |  |  |  |  |  |  |  | 2 | 1 | 1 |  |  |  |  | 4 |
|  | 8 |  |  |  |  |  |  |  |  | 1 | 1 |  |  |  |  | 2 |
|  | 9 |  |  |  |  |  |  |  |  |  | 3 | 2 |  |  |  | 5 |
|  | 10 |  |  |  |  |  |  |  |  |  |  | 3 |  | 1 | 1 | 5 |
|  | 11 |  |  |  |  |  |  |  |  |  |  |  |  |  |  |  |
|  | 12 |  |  |  |  |  |  |  |  |  |  |  |  |  |  |  |
|  | 13 |  |  |  |  |  |  |  |  |  |  |  |  |  |  |  |
| Total | |  | 1 |  |  | 3 | 5 | 7 | 4 | 4 | 5 | 5 |  | 1 | 1 | 36 |

Table S13. Cross-tabulation of OMDS at the time of initial interview versus OMDS at the time of 5^th^-year interview (untreated group, n = 32)

|  | | OMDS at the 5^th^-year point | | | | | | | | | | | | | | Total |
| --- | --- | --- | --- | --- | --- | --- | --- | --- | --- | --- | --- | --- | --- | --- | --- | --- |
|  |  | 0 | 1 | 2 | 3 | 4 | 5 | 6 | 7 | 8 | 9 | 10 | 11 | 12 | 13 |  |
| Baseline OMDS | 0 |  |  |  |  |  |  |  |  |  |  |  |  |  |  |  |
|  | 1 |  |  |  |  |  |  |  |  |  |  |  |  |  |  |  |
|  | 2 |  |  | 2 |  |  |  |  |  |  |  |  |  |  |  | 2 |
|  | 3 |  |  |  | 1 | 2 |  |  |  |  |  |  |  |  |  | 3 |
|  | 4 |  |  |  |  | 5 |  |  |  |  |  |  |  |  |  | 5 |
|  | 5 |  |  |  |  |  | 11 | 3 | 1 |  |  |  |  |  |  | 15 |
|  | 6 |  |  |  |  |  |  | 2 | 1 | 1 | 1 |  |  |  |  | 5 |
|  | 7 |  |  |  |  |  |  |  |  |  |  |  |  |  |  |  |
|  | 8 |  |  |  |  |  |  |  |  | 1 |  |  |  |  |  | 1 |
|  | 9 |  |  |  |  |  |  |  |  |  | 1 |  |  |  |  | 1 |
|  | 10 |  |  |  |  |  |  |  |  |  |  |  |  |  |  |  |
|  | 11 |  |  |  |  |  |  |  |  |  |  |  |  |  |  |  |
|  | 12 |  |  |  |  |  |  |  |  |  |  |  |  |  |  |  |
|  | 13 |  |  |  |  |  |  |  |  |  |  |  |  |  |  |  |
| Total | |  |  | 2 | 1 | 7 | 11 | 5 | 2 | 2 | 2 |  |  |  |  | 32 |

Table S14. Cross-tabulation of OMDS at the time of initial interview versus OMDS at the time of 5^th^-year interview (miscellaneous group, n = 33)

|  | | OMDS at the 5^th^-year point | | | | | | | | | | | | | | Total |
| --- | --- | --- | --- | --- | --- | --- | --- | --- | --- | --- | --- | --- | --- | --- | --- | --- |
|  |  | 0 | 1 | 2 | 3 | 4 | 5 | 6 | 7 | 8 | 9 | 10 | 11 | 12 | 13 |  |
| Baseline OMDS | 0 |  |  |  |  |  |  |  |  |  |  |  |  |  |  |  |
|  | 1 |  |  |  |  |  |  |  |  |  |  |  |  |  |  |  |
|  | 2 |  |  |  |  | 1 | 1 |  |  |  |  |  |  |  |  | 2 |
|  | 3 |  |  |  |  |  | 1 |  |  |  |  |  |  |  |  | 1 |
|  | 4 |  |  |  |  | 1 | 3 | 1 |  |  |  |  |  |  |  | 5 |
|  | 5 |  |  |  |  |  | 10 |  |  |  |  |  |  |  |  | 10 |
|  | 6 |  |  |  |  |  |  | 4 | 2 |  |  |  |  |  |  | 6 |
|  | 7 |  |  |  |  |  |  |  | 3 | 1 |  | 1 |  |  |  | 5 |
|  | 8 |  |  |  |  |  |  |  |  | 2 |  |  |  |  |  | 2 |
|  | 9 |  |  |  |  |  |  |  |  |  |  |  |  |  |  |  |
|  | 10 |  |  |  |  |  |  |  |  |  |  | 1 |  |  |  | 1 |
|  | 11 |  |  |  |  |  |  |  |  |  |  |  |  |  |  |  |
|  | 12 |  |  |  |  |  |  |  |  |  |  |  |  |  |  |  |
|  | 13 |  |  |  |  |  |  |  |  |  |  |  |  |  | 1 | 1 |
| Total | |  |  |  |  | 2 | 15 | 5 | 5 | 3 |  | 2 |  |  | 1 | 33 |

Table S15. Baseline characteristics of patients with HAM/TSP with OMDS 3–6 who had been observed for four years (n = 100)

|  | All patients  (n = 100) | Steroid (**S**) group  (n = 32) | Steroid-  history (**SH**) group  (n = 18) | Untreated (**U**) group (n = 28) | Miscella-  neous (**M**) group  (n = 22) | *p* value | Groups with significant difference ^c)^ |
| --- | --- | --- | --- | --- | --- | --- | --- |
| Sex: Female | 72 (72.0%) | 25 (78.1%) | 9 (50.0%) | 20 (71.4%) | 18 (81.8%) | 0.113 ^a)^ | — |
| Age at baseline (year)^*^ | 61.7 ± 9.5 | 62.8 ± 8.2 | 63.2 ± 9.6 | 60.3 ± 11.7 | 60.6 ± 8.5 | 0.627 ^b)^ | — |
| Age at onset (year)^*^ | 44.9 ± 14.1 | 49.3 ± 13.5 | 44.6 ± 17.5 | 43.0 ± 12.7 | 41.0 ± 13.0 | 0.147 ^b)^ | — |
| Disease duration^*^ (Time from onset to initial interview) | 16.3 ± 10.2 | 14.0 ± 9.2 | 17.2 ± 8.6 | 16.3 ± 10.8 | 18.7 ± 11.9 | 0.402 ^b)^ | — |
| Baseline OMDS^*^ | 5.0 ± 0.8 | 5.0 ± 0.8 | 5.3 ± 0.8 | 4.8 ± 0.9 | 5.0 ± 0.8 | 0.184 ^b)^ | — |
| Rapid progressors^**^ | 17 (17.0%) | 6 (18.8%) | 5 (27.8%) | 3 (10.7%) | 3 (13.6%) | 0.473 ^a)^ | — |

Statistical methods: a) By chi-square test, b) By analysis of variance, c) By Tukey post hoc tests

*Data are expressed as mean ± standard deviation, ** Rapid progressors were defined as those who developed OMDS 5 or above within 2 years from the onset of motor symptoms. OMDS, Osame motor disability score

Table S16. Changes in OMDS in patients with HAM/TSP with OMDS 3–6 who had been observed for four years (n = 100)

|  | Baseline* | 2^nd^-year point^*^ | 3^rd^-year point^*^ | 4^th^-year point^*^ | 5^th^-year point^*^ | ∆OMDS per four years** | *p* value ^†^ |
| --- | --- | --- | --- | --- | --- | --- | --- |
| All patients | 4.98 ± 0.83 | 5.13 ± 0.98 | 5.32 ± 1.19 | 5.41 ± 1.27 | 5.53 ± 1.31 | 0.55 (0.37–0.73) | <0.001 |
| Steroid group | 4.97 ± 0.78 | 5.16 ± 1.02 | 5.44 ± 1.39 | 5.56 ± 1.54 | 5.72 ± 1.63 | 0.75 (0.34–1.16) | 0.001 |
| Steroid-history group | 5.33 ± 0.77 | 5.44 ± 0.86 | 5.61 ± 0.98 | 5.72 ± 1.02 | 5.83 ± 1.15 | 0.50 (0.11–0.89) | 0.015 |
| Untreated group | 4.79 ± 0.88 | 4.93 ± 1.05 | 5.14 ± 1.27 | 5.18 ± 1.36 | 5.25 ± 1.32 | 0.46 (0.16–0.77) | 0.004 |
| Miscellaneous group | 4.95 ± 0.84 | 5.09 ± 0.92 | 5.14 ± 0.89 | 5.23 ± 0.81 | 5.36 ± 0.73 | 0.41 (0.11–0.70) | 0.009 |

*Data are expressed as mean ± standard deviation, **Data are expressed as point estimates and 95% confidence intervals, † Statistical methods used a paired t-test (baseline vs. 5^th^-year point)
