# Supplementary material for: Real-world clinical course of HTLV-1-associated myelopathy/tropical spastic paraparesis (HAM/TSP) in Japan
Source: Orphanet J Rare Dis. 2019 Oct 21;14:227. doi: 10.1186/s13023-019-1212-4 (PMC6802124; doi:10.1186/s13023-019-1212-4)
Supplement: Supplementary file 3 — Additional file 3: Figure S2. Chronological change in OMDS in the four-year observation group (n = 148). Each bar in this bar chart represents 148 patients (four-year observation group, Fig. 2e) as 100% and indicates the percentage of patients belonging to each OMDS at each survey time. [file 13023_2019_1212_MOESM3_ESM.docx]

**Additional file 3**

Figure S2. Chronological change in OMDS in the four-year observation group (n = 148)

Each bar in this bar chart represents 148 patients (four-year observation group, Figure 2e) as 100 percent and indicates the percentage of patients belonging to each OMDS at each survey time.
